# Tunnel-Ventilated Sheds with Negative Pressure Reduce Thermal Stress and Improve the Meat Quality of Broilers

**DOI:** 10.3390/ani14142017

**Published:** 2024-07-09

**Authors:** Karina Suemi Sakamoto, Robson Mateus Freitas Silveira, Natália Cristina Benincasa, Carmen Josefina Contreras Castillo, Cristian Marcelo Villegas Lobos, Iran José Oliveira da Silva

**Affiliations:** 1 Department of Biosystems Engineering, Luiz de Queiroz College of Agriculture, University of São Paulo (ESALQ/USP), Piracicaba 13418-900, SP, Brazil; karinasakomoto@usp.br (K.S.S.); nataliabenincasa@usp.br (N.C.B.); iranoliveira@usp.br (I.J.O.d.S.); 2 Department of Animal Science, Luiz de Queiroz College of Agriculture, University of São Paulo (ESALQ/USP), Piracicaba 13418-900, SP, Brazil; 3 Department of Agrifood Industry, Food and Nutrition, Luiz de Queiroz College of Agriculture, University of São Paulo (ESALQ/USP), Piracicaba 13418-900, SP, Brazil; ccastill@usp.br; 4 Department of Exact Science, Luiz de Queiroz College of Agriculture, University of São Paulo (ESALQ/USP), Piracicaba 13418-900, SP, Brazil; clobos@usp.br

**Keywords:** transport, heat stress, PSE meat, tropical climate

## Abstract

**Simple Summary:**

Positive and negative pressure ventilation systems are widely used in poultry farming to control the internal environment of poultry houses, especially in tropical regions, in which hot and humid climates can pose challenges to the thermal regulation of animals. Knowing that thermal stress impacts the quality of animal meat, this study aimed to evaluate the thermal performance and quality of poultry meat in these two types of shed. The main results found here were that the negative pressure system presents lower temperatures and air enthalpy, and the animals reared in this system have better quality meat. We recommend the negative ventilation system for broiler producers in tropical regions due to its ability to provide better thermal indicators and chicken meat quality.

**Abstract:**

This study aimed to evaluate the thermal performance and meat quality in broilers reared in positive pressure tunnel ventilation (PP) and negative pressure tunnel ventilation (NP) in production houses. 320 Cobb broilers (40 broilers per house) were used. *Pectoralis major* muscles from 40 broilers (10 broilers per house) were randomly selected and analysed for L* (lightness), a* (redness), b* (yellowness), pH, drip loss (DL), cooking loss (CL) and shear force (SF). Air temperature and humidity of the transportation and slaughterhouse waiting room were recorded in the last week of rearing. Subsequently, the enthalpy comfort index (ECI) was calculated. Air temperature and ECI were higher (*p* < 0.05) in positive pressure sheds, whereas relative humidity was higher (*p* < 0.001) in negative pressure sheds. There was no statistically significant difference between the enthalpy comfort index during transport and lairage (*p* > 0.005). Meat quality defects (high L*, DL, CL, SF) were found in PP and NP. It was observed that b* was higher in PP, although pH and CL were higher in NP. Differences in pH, b* and CL indicate that broilers from PP had a higher level of heat stress. In conclusion, differences in pH, b*value and cooking loss in breast broilers indicate that birds in PP had a higher level of heat stress. Additional studies investigating pre-slaughter handling methods to minimise injuries and heat stress are recommended in order to improve animal welfare and meat quality.

## 1. Introduction

The rearing of broiler chickens depends on resources, and it currently takes several weeks for birds to reach the body weight and carcass quality that make them suitable for slaughter. However, all this effort can be lost within a few hours before slaughter if pre-slaughter operations are not well conducted, resulting in damage to the welfare of birds, losses in carcass value and meat quality defects [1].

During the pre-slaughter period, broilers are subject to potential stressors such as food and water deprivation, handling, disruption of social groups, high crate density, thermal discomfort, vibration during transport, noise and unfamiliar environments. These situations are closely related to poor animal welfare and production losses, influencing mortality rate, musculoskeletal disorders, cardiovascular diseases, increased susceptibility to stress, injuries and meat quality defects [2,3].

The concept of chicken meat quality is a multifactorial issue that involves genetics, nutrition, management, slaughter procedures, meat processing and storage. However, the effects of pre-slaughter and slaughter management may be more significant than variation in management practices during the rearing period. It can decrease carcass yield, increase the presence of lesions and microbial contamination, and cause defects in meat quality, such as creating pale, soft and exudative (PSE) meat and dark, firm and dry (DFD) meat [4]. Therefore, failures in animal welfare can indirectly impair the meat quality of these birds [5].

Ventilation systems control the indoor environment (air temperature, relative humidity, litter moisture and ammonia) and are crucial for broiler production and welfare [6]. Houses with a positive pressure ventilation system use fans which push air into the building, which creates a higher positive pressure. On the other hand, houses with a negative pressure ventilation system use exhaust fans, which create negative pressure and pull air outside through inlets. The main disadvantage of the negative ventilation system is the thermal environment because in this system there is no refrigeration system, only ventilation, which does not reduce the temperature, changing only the heat sensation and not actually a achieving a reduction in temperature.

Aviaries with a negative pressure ventilation system provide greater thermal comfort and better growth performance for birds as air is extracted, creating a lower pressure inside the shed compared to outside. This draws fresh air from the evaporative pad through controlled inlets. Nascimento et al. [7] reported that negative ventilation systems presented higher air velocities compared to conventional ventilation (positive pressure) systems and that they enabled greater transfer of sensible heat by the birds, suggesting better thermal comfort conditions. It is also known that thermal stress influences the quality of poultry meat [8], but there are few studies evaluating how meat quality depends on the type of facilities the animals experience, especially in tropical regions. Our hypothesis is that chickens reared in a negative pressure barn system have higher meat quality under better thermal conditions.

The objective of this study was to evaluate the thermal performance and meat quality of broiler chickens reared in positive pressure ventilation tunnels (PP) and negative pressure ventilation tunnels (NP) in tropical regions.

## 2. Materials and Methods

Animal experimental procedures in this research were carried out from June to September 2016. The research was approved by the Ethics Commission for Animal Use (CEUA), protocol n^o^. 2016–04, of Luiz de Queiroz College of Agriculture—University of São Paulo, Piracicaba, São Paulo, Brazil.

**Figure 1:**
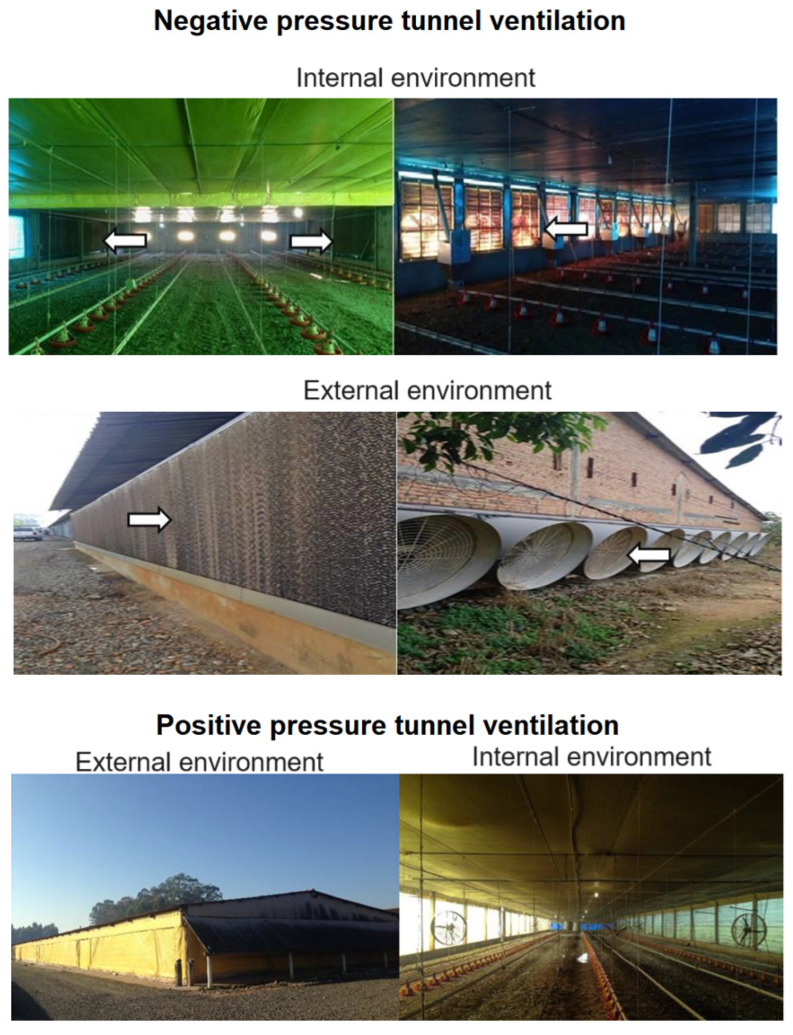
Internal and external views of the experimental sheds.

The research was carried out in four commercial broiler houses, two positive (PP) and two negative (NP) pressure tunnel ventilation systems in Tietê, São Paulo, Brazil (Latitude: 23°06′07″ S; Longitude: 47°42′53″ W; Altitude: 508 m). 78,000 birds were housed in the four sheds (320 broiler chickens were randomly selected for meat quality analysis [(40 broilers per house)]. The PP had longitudinal fans distributed throughout the entire house. NP had exhaust fans located on one end of the house and evaporative cooling pads at the other end. PP and NP were equipped with a fogging system, automatic drinkers and feeders. The four commercial broiler houses were part of a vertically integrated chicken meat company, which provides chicks, feed, medication, supervision and transport (Figure 1).

The average final animal density was 34.51 ± 2.02 kg/m^2^ for PP or 32.50 ± 1.71 kg/m^2^ for PN. At the end of the production cycle, 80 Cobb mixed-sex broilers (50% male and 50% female; 2.61 ± 0.19 kg average body weight at 44 days old) from each commercial broiler house were randomly selected (320 broilers in total for the study). The fasting period was 8 h. During the night-time, the catching team caught the birds by the neck in an upright position and placed them in transport crates (density per crate 536.8 cm^2^/broiler) at the rear of the trucks. Short-distance (up to 50 km) shipments were carried out between midnight and 3 am. At the slaughterhouse, the trucks with the broilers were lairaged in an acclimatising holding area with fans and foggers. Average waiting time was 5.43 h in the PP and 4.33 h in the NP.

### 2.1. Enthalpy Comfort Index (ECI)

The microclimate condition was assessed by calculating the Enthalpy Comfort Index (ECI) during transport and lairage. Each transport crate (40 crates in total for the study) was fitted with data loggers (U12-011, HOBO^®^, Melrose, MA, USA). Air temperature (AT) and air relative humidity (RH) were measured for each transport crate every minute during transport and lairage. Air temperature (AT) and relative humidity (RH) were used to calculate ECI.

ECI = {1.006 × AT + [(RH/BP) × 10 (7.5 × AT/237.3 + AT)]} × (71.28 + 0.052 × AT)(1)

In which: ECI = Enthalpy Comfort Index (KJ Kg^−1^ dry air); AT = Air temperature (°C); RH = Relative Air Humidity (%); BP = Barometric pressure (mmHg);

ECI was classified into a comfort zone (35.0 to 48.0 kJ.kg^−1^ of dry air), warning zone (48.1 to 57.6 kJ.kg^−1^ of dry air), and critical zone (57.7 to 66.1 kJ.kg^−1^ of dry air) for broilers over 42 days old.

### 2.2. Meat Quality Evaluations

After the chilling process had taken place, ten *Pectoralis major* muscle (forty breasts in total for the study) were randomly selected from each broiler house, individually vacuum-packed in plastic bags, and stored in a dark chilled room at 4 ± 1 °C. Left breast fillets were used to evaluate colour, pH and drip loss. On the other hand, right breast fillets were used to evaluate cooking loss and shear force. Colour, pH, drip loss and cooking loss were analysed 24 h after slaughter; and shear force after 48 h after slaughter.

#### 2.2.1. Colour Measurements

The colour on the surface of breast fillets was performed using a colourimeter (CR-400 Model Chroma Meter, Konica Minolta^®^, Mahwah, NJ, USA). The parameters L* (lightness), a* (redness) and b* (yellowness) were recorded based on the International Commission on Illumination (CIE) System. The colourimeter was calibrated using standard white porcelain (Y = 93.7, x = 0.3160 and y = 0.3323). Three scans from each breast fillet were recorded and averaged for statistical analysis.

#### 2.2.2. pH Measurements

The pH was determined with a calibrated potentiometer (pH 1140 model, Mettler-Toledo, Switzerland) with automatic temperature compensation and a glass penetration electrode (Digimed, Presidente Prudente, São Paulo, Brazil) at three randomly selected localisations in individual breast fillets.

#### 2.2.3. Drip Loss Measurements

Drip loss was determined using the Honikel bag method. Each left breast fillet sample (about 100 g) was suspended inside plastic bags, ensuring that the sample did not touch the sides of the bag, at 2 ± 1 °C for 72 h. Drip loss was expressed as a percentage of the weight loss in 72 h compared with the initial sample weight.

#### 2.2.4. Cooking Loss Measurements

Right breast fillets were weighed and cooked individually in vacuum-packed plastic bags immersed in a water bath at 85 °C for 30 min [9]. The exudates were drained, and the cooked samples were chilled and stored at 2 ± 1 °C for 24 h, then reweighed. Cooking loss was expressed as a percentage of the weight loss in 24 h, compared with the initial sample weight.

#### 2.2.5. Shear Force Measurements

After cooking loss measurements, the right breast fillets were used for shear force analysis. Samples were cut into 3 rectangular blocks of a 1 cm^2^ cross-section, with the fiber direction parallel to a long dimension of 2 cm. Shear force was determined using a Texture Analyser (TA.XT2i Stable Micro Systems Ltd., Surrey, UK) equipped with a Warner Bratzler shear blade with a 50 kgf load cell applied at a cross-head speed of 1 mm.s^−1^ [10]. Samples were cut perpendicular to the fiber direction. Shear force was calculated as the average shear force from the 3 samples.

### 2.3. Classification of the Sample into Quality Groups

The combination of pH and L* analyses were used to classify the samples of broiler breast muscle in DFD (pH > 6.1 and L* < 46.0), normal (5.7 ≤ pH ≤ 6.1 and 46.0 ≤ L* ≤ 53.0) and PSE (pH < 5.7 and L* > 53.0) groups as recommended by Barbut et al. [11] and Dadgar et al. [12].

### 2.4. Statistical Analysis

For evaluation of the enthalpy comfort index during transport and lairage, we used the Kruskal-Wallis test.

For the quantitative variables L*, a*, b*, pH and shear force, we used a completely randomised design for comparing PP and NP. For the pH variable, we used the Box and Cox [13] transformation due to the original variable not holding the assumption of the model (errors normally distributed and error variance constant). On the other hand, for the percentage variables (drip loss and cooking loss), we used a beta regression model with logit link function [14] for comparing PP and NP. The term Z, in our results, represents the value of a statistic that has a normal standard distribution.

## 3. Results

### 3.1. Thermal Performance

Air temperature and ECI were higher (*p* < 0.05) in positive pressure sheds, while relative humidity was higher (*p* < 0.001) in negative pressure sheds (Table 1). There was no statistically significant difference between the enthalpy comfort index during transport (*p* > 0.05) and lairage (*p* > 0.0001). The value of the enthalpy comfort index was similar between types of shed during transport and accommodation (Table 2).

### 3.2. Meat Quality

The analyses of L* (F (1,38) = 1.611, *p* = 0.212), a* (F(1,38) = 0.008, *p* = 0.927), drip loss (Z = −0.833, *p* = 0.405) and shear force (F(1,38) = 0.149, *p* = 0.700) of the *Pectoralis major* muscle did not show differences between PP and NP (Table 2). It was observed that b* (F(1,38) = 9.673, *p* = 0.003) was higher in broilers reared in PP than NP, although pH (F(1,38) = 20.044, *p* < 0.0001) and cooking loss (Z = −6.569, *p* < 0.0001) were higher in birds from NP than PP.

Within NP, negative correlations were observed between L* and pH (r = −0.514, *p* = 0.021) and between pH and drip loss (r = −0.471, *p* = 0.036) (Table 3). In addition to NP, there were positive correlations between L* and shear force (r = 0.498, *p* = 0.025), and between L* and drip loss (r = 0.665, *p* = 0.001). Within PP, a negative correlation was found between b* and shear force (r = −0.681, *p* = 0.001), while a positive correlation was found between shear force and cooking loss (r = 0.455, *p* = 0.044) (Table 4 and Table 5).

## 4. Discussion

### 4.1. Thermal Performance

The lower ECI indices of the negative pressure shed are mainly explained by the lower air temperature, a variable which is included in the enthalpy calculation. Based on the enthalpy values, it can still be stated that the birds were submitted to a thermal comfort zone in the negative pressure shed, and submitted to thermal stress in the positive pressure shed, according to the Broiler Enthalpy Tables [15]. It is already agreed in the literature that positive pressure sheds present a lower condition of thermal comfort and well-being for birds when compared to negative pressure sheds; this is mainly justified because of the cooling system of the sheds (Figure 1), where in negative pressure systems the thermal environment is better controlled.

### 4.2. Meat Quality Evaluation

PSE or DFD meat was not observed in PP and NP. However, meat quality defects and differences between chicken meat characteristics (b*, pH and cooking loss) were found between PP and NP.

In both broiler houses, L* values were classified as high (L* > 53.0). In PP and NP, the birds were raised and slaughtered during winter and early spring; despite this, the L* values were characteristic for summer months, where birds tend to have paler meat due to heat stress [16]. The high L* values can probably be explained by the birds’ thermal discomfort during lairage [17]. ECI was classified as within the warning zone for PP and the critical zone for NP during lairage. Heat stress can result in glycolytic changes and denaturation of sarcoplasmic proteins. It increases the amount of light scattering, and therefore higher L* values [17]. Moreover, it can cause lower values of a* due to the greater oxidation of myoglobin in the muscles of birds exposed to heat stress [18].

It was observed that b* values were higher in chickens raised in PP than NP. According to Warris [19], a high b* value was observed in chicken meats with a high L* value and low pH. The pH values were lower in PP than NP. The pH values were within the normal range, although the birds had suffered heat stress in PP and NP. This finding was also found by Wang et al. [20]. Thus, the higher b* values in PP may be due to high L* values in meat due to heat stress.

Water holding capacity (WHC) is a critical issue in the poultry industry as it is related to product yield. There was no difference in drip loss values between PP and NP, but drip loss values were considered high. Wang et al. [20] reported that heat stress could accelerate pH drop under commercial conditions and increase drip loss. Birds exposed to heat stress had an increased metabolic rate during rigor mortis, leading to severe protein denaturation and low WHC of meat [21]. Higher cooking loss values were observed in NP than in PP. In both broiler houses, cooking loss values were higher than in a similar study. Dos Santos et al. [22] found that broilers exposed to heat stress within a warning zone during short-distance transport presented 15.59% cooking loss. The birds from the NP were under heat stress during transport and lairage, and were classified, respectively, as within the worrying and critical zones [23]. Moreover, birds in PP suffered heat stress classified as within the warning zone during lairage. The duration and heat stress during transport and lairage, especially in NP, can probably explain the high cooking loss values.

Meat tenderness is one of the main factors attributed to consumer acceptance. Shear force assesses the tenderness of the meat. There was no difference in shear force values between PP and NP, but broiler breasts were considered less tender than they were in other studies. Thielke et al. [24] found shear force values for broiler breasts after 24 h before freezing between 2.37 and 2.43 kgf. However, the results of this study corroborate other studies, which reported average values of 4.79 kgf for L* > 60 [25]. The high drip loss and cooking loss values of both broiler houses probably resulted in a decrease in the tenderness of the chicken breasts.

Within NP, negative correlations were observed between L* and pH, and between pH and drip loss, in agreement with studies reported previously [22,26]. Positive correlations were found between L* and shear force, and between L* and drip loss [26,27]. Low pH values cause protein denaturation, which leads to an increase in light reflectance, poor WHC and exudation. It results in high values of L*, drip loss and shear force.

Within PP, a negative correlation was observed between b* and shear force; however, this correlation was not found by Dos Santos et al. [22]. Broiler breasts in PP showed high b* values due to high L* values [19]. Besides, meat colour may change by scalding or by cooling in the chiller during industrial processing [28,29]. Shear force showed a positive correlation with cooking loss, as found by Dos Santos et al. [22] since poor WHC increases cooking loss and leads to an increase in shear force.

Fast-growing broiler strains may have influenced meat quality parameters in this study. Rapidly growing birds are selected for high breast yield and may experience a loss in thermoregulatory capacity compared to precursor strains. They may be more susceptible to thermal stress during the pre-slaughter period and, consequently, present muscle problems, metabolic disorders and reduced meat quality [30,31]. Also, this demonstrates the importance of efficient environmental control mechanisms during lairage, and highlights the influence of bioclimatic conditions on meat quality.

It was demonstrated in this study that the generation of injuries and meat quality can be affected by multiple factors that interact with each other in a complicated way. This shows the importance of working together with all the areas involved to obtain a high-quality product. The market has established requirements and specifications for food quality and animal welfare. This means that animal production is expected to become more efficient, with an increased focus on animal husbandry and working conditions for employees in the chain [3].

In conclusion, birds reared in negative pressure poultry production systems have better meat quality in tropical conditions.

## Figures and Tables

**Table 1:** Environmental variables and specific enlthalpy index of the different sheds.

Variables	Positive Pressure Tunnel Ventilation	Negative Pressure Tunnel Ventilation	*p*-Value
Air temperature (%)	26.58 ± 0.48 ^a^	22.72 ± 0.80 ^b^	<0.0001
Relative humidity (%)	67.70 ± 2.57 ^b^	72.87 ± 3.01 ^a^	<0.0001
ECI (kJ.kg^−1^ of dry air)	60.14 ± 2.46 ^a^	51.75 ± 4.24 ^b^	=0.024

^ab^ Different letters in lines identify means that are statistically distinct according to the Kruskal-Wallis test at 5% significance.

**Table 2:** Enthalpy Comfort Index (ECI) during transport and lairage in PP and NP (Mean ± SD (kJ.kg^−1^ of dry air).

ECI	Negative Pressure Tunnel Ventilation	Positive Pressure Tunnel Ventilation	*p*-Value
Transport	45.66 ± 2.93 ^a^	50.05 ± 7.78 ^a^	0.145
Lairage	55.15 ± 10.93 ^a^	63.00 ± 12.06 ^a^	0.541

^a^ Equal letters in lines identify means that are statistically distinct according to the Kruskal-Wallis test at 5% significance.

**Table 3:** Meat quality of broilers reared in different types of shed.

Variables	Poultry Houses ^c^		*p*-Value
	Negative Pressure Tunnel Ventilation	Positive Pressure Tunnel Ventilation	
L*	60.7 ± 3.86	62 ± 2.56	=0.547
a*	2.26 ± 1.08	2.30 ± 1.35	=0.721
b*	4.64 ± 2.30 ^b^	7.57 ± 3.46 ^a^	<0.001
pH	6.18 ± 0.20 ^a^	5.91 ± 0.21 ^b^	=0.012
Shear force	4.40 ± 1.64	4.58 ± 1.94	=0.351
Cooking loss	27 ± 3.92 ^A^	18.6 ± 4.02 ^B^	=0.001
Drip loss	3.72 ± 1.50	3.34 ± 1.51	=0.245

^a,b^ Different letters in lines identify means that are statistically distinct according to the completely randomised design at 5% significance. For pH, the Box and Cox (1964) transformation was used; ^A,B^ Different letters in lines identify means that are statistically distinct according to the beta regression model with logit link function at 5% of significance; ^c^ Mean ± SD, n = 20 per group; L* (lightness); a* (redness); b* (yellowness), SF (shear force), CL (cooking loss), DL (drip loss).

**Table 4:** Pearson correlation coefficients for negative pressure tunnel ventilation shed between meat quality variables (L*, a*, b*, pH, SF, CL and DL).

	Meat Quality Variables					
	a*	b*	pH	SF	CL	DL
L*	0.012	0.323	−0.514 *	0.498 *	−0.196	0.665 ***
a*	-	0.060	−0.090	0.035	0.333	0.003
b*	-	-	−0.219	−0.131	0.245	0.046
pH	-	-	-	−0.257	−0.012	−0.471 *
SF	-	-	-	-	0.212	−0.060
CL	-	-	-	-	-	−0.302

*** *p* < 0.001; * *p* < 0.05; Note: L* (lightness); a* (redness); b* (yellowness), SF (shear force), CL (cook loss), DL (drip loss).

**Table 5:** Pearson correlation coefficients for positive pressure tunnel ventilation shed between meat quality variables.

	Meat Quality Variables					
	a*	b*	pH	SF	CL	DL
L*	−0.078	−0.206	−0.265	0.342	−0.43	−0.063
a*	-	0.399	0.318	−0.202	0.28	−0.14
b*	-	-	0.335	−0.681 ***	−0.39	−0.282
pH	-	-	-	−0.257	−0.142	−0.012
SF	-	-	-	-	0.455 *	0.185
CL	-	-	-	-	-	0.102

*** *p* < 0.001; * *p* < 0.05; Note: L* (lightness); a* (redness); b* (yellowness), SF (shear force), CL (cook loss), DL (drip loss).

## Data Availability

Data will be made available upon request to the corresponding author.
